# Will a mAb-Based Immunotherapy Directed against Cancer Stem Cells Be Feasible?

**DOI:** 10.3389/fimmu.2017.01509

**Published:** 2017-11-09

**Authors:** Silvia Santamaria, Marisa Delgado, Leonor Kremer, Jose A. Garcia-Sanz

**Affiliations:** ^1^Cancer Genetics and Cancer Stem Cell Laboratory, Centro de Investigaciones Biologicas, Department of Cellular and Molecular Medicine, Spanish National Research Council (CSIC), Madrid, Spain; ^2^Centro Nacional de Biotecnologia, Department of Immunology and Oncology, Spanish National Research Council (CSIC), Madrid, Spain

**Keywords:** cancer stem cells, therapeutic antibodies, immunotherapy, effective cancer therapies, cancer genetics

## Abstract

The cancer stem cell (CSC) hypothesis suggests that within a tumor, there is a small subpopulation of cells with stem cell properties responsible for tumor maintenance and metastasis generation. This hypothesis also implies that new antitumor drugs, rather than targeting the bulk of the tumor mass, would be more effective if they directly targeted the CSC subpopulation. The CSCs from several types of tumors have been identified with mAbs recognizing surface antigens in these cells; however, antigens specifically or exclusively expressed in the CSC population have not yet been identified. Thus, questioning the possibility of using therapeutic antibodies directed against the CSCs. Here, we review the possibilities of using antibodies directly targeting the CSCs as therapeutic agents in the form of naked antibodies, antibodies conjugated to nanoparticles, or antibody cocktails.

## Introduction

Although the most frequently used anticancer treatments still are chemotherapy and radiotherapy, it is clear by now that monoclonal antibodies have emerged on the last 20 years as the most relevant new type of anticancer drugs with clinically proven therapeutic value. Concomitantly, this has generated an enormous interest, which has led to a burst of new approaches and clinical trials, where monoclonal antibodies represent the key element (1). However, most of the current anticancer treatments, including antibodies or other molecular interventions, increase the survival and improve the quality of life on patients, but do not necessarily cure.

It is obvious that antibodies against HER2, CD20, VEGF, EGFR, or CD52 have shown their clinical therapeutic value as anticancer drugs (1). In addition, antibodies that enhance the immune response by either blocking the PD-1/PD-L1 axis (2); antibodies anti-CTLA-4 (3, 4); or antibodies that block inhibitory receptors of NK cells (5, 6); or even CAR T cells (variable antibody regions engineered TCR-carrying T cells) (7), have proven also very useful. Indeed, they are able to redirect the antitumor immune response and allow envisaging the possibility of a cure for cancer patients. Obviously, the cure for cancer patients might come from the use of more or less complex combinations of antibodies that will include other drugs or cells (8).

Thus, the remaining questions are as follows: *Is this the best we can do to cure cancer patients? Are we hitting the right targets?* In this review, we would like to discuss the characteristics of the cancer stem cells (CSCs) that make them ideal targets, and the possible strategies of using antibodies to directly target the CSC population as the best option to cure cancer patients.

## Adult Stem Cells and CSCs

**Figure 1:**
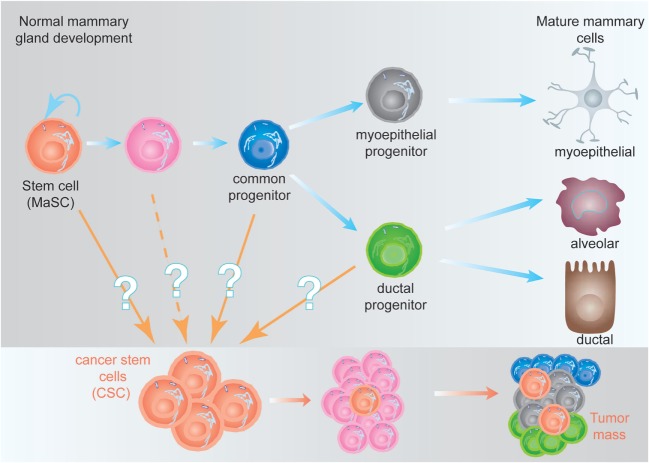
Hypothetical model of the mammary epithelial hierarchy and its relationship with cancer stem cells (CSCs). (Top) The mammary stem cell (MaSC) differentiates through a common progenitor into either a myoepithelial or a ductal progenitor, which are committed to generate mature myoepithelial or ductal and alveolar cells, respectively. During this process, the MaSC and its progeny undergo at least nine cell divisions to generate the fully differentiated cells (not represented here), giving a ratio 1:500 MaSC:differentiated cells (18). (Bottom) CSCs, independent of their origin, are malignant-transformed cells with stem cell characteristics. They are able to generate a tumor (or metastases), although they represent a small fraction of the tumor mass (9).

One of the concepts that have largely changed our understanding about tumor biology was the CSC hypothesis (9). Stem cells are defined as cells with the ability of self-renew (perpetuate themselves) and to differentiate, generating mature cells of a particular tissue. Adult (or tissue-specific) stem cells are rare cells that have been identified in many tissues, including the hematopoietic stem cells (HSCs) in the bone marrow (10, 11), the mammary stem cells in the mammary gland (12, 13), neural stem cells in the nervous system (14, 15), and the intestine stem cells in the intestine (16), among others. In several cases, a hierarchical structure has been demonstrated, where adult stem cells generate the appropriate cells from that tissue and maintain its homeostasis. The adult stem cell is able to undergo either symmetric cell divisions, generating two daughter stem cells, or asymmetrically, where the stem cell gives rise to a daughter stem cell and another cell committed for differentiation (17). From the committed cell, a common progenitor will be generated lacking self-renewal ability, but able to generate all the cell types of the differentiated tissue. The common progenitor will in turn generate more committed progenitors; each one of them will be able to generate one or two differentiated cell types from the tissue (Figure 1). This differentiation process is concomitant with cell expansion, explaining the reason why in many cases the frequency of adult stem cells is below 1% (18).

The CSC hypothesis proposes for tumors a hierarchical structure similar to the described for adult tissues. A small fraction of cells within the tumor harbor stem-cell like characteristics (referred to as CSCs), with an indefinite self-renewal potential and able to drive tumorigenesis, being able to develop into a heterogeneous, more differentiated population, which constitutes the tumor mass (9). The CSCs were initially identified in acute myeloid leukemia (19) and prospectively identified in solid tumors including the mammary gland (20), the brain (21), and many others. The existence of CSC has been unequivocally demonstrated *in vivo* in glioblastomas, intestine, melanomas, and mammary tumors (22–25). One of the predictions of the CSC hypothesis was that more effective cancer therapies would target the CSC, instead of the bulk of the tumor (9). This is supported by the finding that CSC, such as normal stem cells, are more resistant to conventional chemotherapy and radiotherapy than more differentiated tumor cells (26), suggesting that effective therapies against the CSC would target self-renewal and/or differentiation of these cells (27).

Interestingly, it has been demonstrated in glioblastomas that therapies directly targeting the CSC are more effective than the ones targeting the tumor mass. In fact, standard chemotherapy was able to kill the bulk of the glioblastoma, but not the CSC, and the tumors quickly returned. When, in addition to chemotherapy, the CSC population was depleted in mouse glioblastoma models using a genetic trick, the tumors shrank back into “residual vestiges” that did not resemble glioblastomas (22). Thus, these data suggest that the predictions of the CSC hypothesis are true and that therapies directed to the CSC will turn out to be more effective.

## CSC Markers

Once established that the CSC represents a distinct tumor cell population, involved in tumor formation and maintenance, the identification of their specific markers has been a priority. First, for the isolation of the CSC and a more detailed analysis on their biology, but also for the possibility of using some of these markers as putative therapeutic targets. In many cases, the combination of positive and negative expression of surface markers allowed the identification of CSC populations. For example, on the identification of CSC in acute myelocytic leukemia (AML), where the cells were fractionated on the basis of CD34 and CD38 expression, demonstrating that only the CD34^+^CD38^−^ cells, but not the CD34^+^CD38^+^ or CD34^−^ cells, were able to engraft immunocompromised mice, replicating many aspects of human AML (19). Similarly, combinations of other surface markers, such as CD24, CD44, ESA, and CD133, allowed the identification of CSC in tumors from breast (20), liver (28), brain (21), lung (29), colon (30), prostate (31), pancreas (32, 33), head and neck squamous carcinoma (34), multiple myeloma (35), melanoma (36), among others (Table 1). It should be noted that in many cases, the surface markers used to identify CSC also identify adult stem cells on the corresponding normal tissues, or are surface markers shared by other cell types (Table 2).

**Table 1:** Phenotypic markers used to identify cancer stem cell (CSC).

Tumor type	Phenotype of CSC	Reference
Brain	CD133^+^	(21, 37)
	CD133^+^ BCRP1^+^A2B5^+^SSEA-1^+^	(38–41)
Breast	CD44^+^CD24^−/low^ESA^+^ALDH-1^high^	(20, 42)
Colon	CD133^+^CD44^+^CD166^+^EpCAM^+^CD24^+^	(43–45)
Head and neck	CD44^+^	(34)
Kidney	CD105^+^	(46)
Leukemia	CD34^+^CD38^−^HLA-DR^−^CD71^−^CD90^−^CD117^−^CD123^+^	(47)
Liver	CD133^+^CD49f^+^CD90^+^	(48, 49)
Lung	CD133^+^ABCG2^high^	(50, 51)
	CD133^+^Sca1^+^CD45^−^PECAM^−^CD34^+^	(52)
	Musahi-1^+^2^+^CD34^+^CD21^+^cKIT^+^p63^+^OCT-4^+^	(53)
Melanoma	CD20^+^	(36)
	CD133^+^CD166^+^Nestin^+^	(54)
Multiple myeloma	CD138^−^	(35, 55)
Ovarian	CD133^+^	(56)
	CD133^+^CD117^+^CD44^+^CD24^+^ALDH1A1^+^	(57, 58)
Pancreas	CD133^+^CD44^+^EpCAM^+^CD24^+^	(59, 60)
Prostate	CD133^+^CD44^+^α2β1^high^	(31)
Retinoblastoma	CD44^+^CD133^−^CXCR4^−^CD90^−^	(61)

**Table 2:** Distribution of frequently used cancer stem cell phenotypic markers.

Phenotypic marker	Tumor type	Reference^a^	Normal tissue expression^b^
CD133^+^	Brain, liver, lung, colon, prostate, pancreatic, and ovary	(21, 31, 37–41, 43–45, 48–52, 54, 56, 59, 60)	1, 2, 4, 5, 6, 7, 8, 11, 12, 14, 16, 17, 18, 19, 20, 21, 22, 23, 26, 27
ESA1	Breast	(20, 42)	All tissues high
CD44^+^	Breast, colon, prostate, pancreas, and head and neck	(20, 31, 34, 42–45, 59, 60)	5, 10, 11, 16, 19, 20, 22, 24, 26, 27
EpCAM^+^	Colon and pancreatic	(43–45, 59, 60)	1, 2, 4, 5, 6, 8, 11, 12, 13, 14, 15, 16, 18, 19, 20, 21, 22, 26, 27
CD20	Melanoma	(36)	1, 3, 5, 6, 8, 9, 10, 11, 12, 14, 16, 18, 19, 20, 22, 23, 26, 27
CD49f^+^	Breast and liver	(20, 42, 48, 49)	1, 8, 12, 14, 15, 16, 17, 27
CD34^+^	Leukemia	(47)	5, 15, 16, 17, 19, 20, 21, 23
CD123^+^	Leukemia	(47)	5, 10, 11, 19, 20
CD24^+^	Colon and pancreatic	(43–45, 59, 60)	n.a.^c^
BCRP1^+^	Brain	(38–41)	n.a.
ABCG2	Lung	(50, 51)	1, 3, 5, 6, 7, 12, 14, 16, 17, 19, 21, 23, 25, 27
CD138^+^	Multiple myeloma	(35, 55)	1, 2, 3, 4, 5, 6, 8, 9, 10, 11, 12, 13, 14, 17, 19, 20, 21, 22, 24, 27
CD90^+^	Liver	(48, 49)	1, 2, 3, 4, 5, 6, 7, 8, 9, 10, 11, 12, 13, 14, 15, 16, 17, 18, 19, 20, 21, 22, 23, 24, 25, 27
CD166^+^	Colon	(62)	5, 7, 21, 25

*^a^Reference on the expression of the phenotypic marker in different tumor types*.

*^b^Each number corresponds to a normal tissue with expression levels >10-fold over background. The data have been obtained from Fagerberg et al. (63). The code number for each tissue is as follows—1: colon; 2: kidney; 3: liver; 4: pancreas; 5: lung; 6: prostate; 7: brain; 8: stomach; 9: spleen; 10: lymph node; 11: appendix; 12: small intestine; 13: adrenal gland; 14: duodenum; 15: adipose tissue; 16: endometrium; 17: placenta; 18: testis; 19: gall bladder; 20: urinary bladder; 21: thyroid gland; 22: esophagus; 23: heart; 24: skin; 25: ovary; 26: bone marrow; and 27: salivary gland*.

*^c^n.a.: data for protein expression of this gene in normal tissues are not available in reference (63)*.

The available data allow raising the question of whether there are specific CSC markers. Although at this time we cannot formally exclude their existence, since the CSC possess the same genetic information as the rest of the tumor (there are no additional mutations in the CSC as compared to the tumor mass), it is more likely that the phenotypic differences on CSC are due to differential gene expression. Indeed, both phenotypic and genetic analyses have failed so far, to pinpoint a single marker specific of any CSC population. In this context, genetic analyses aiming to understand self-renewal, a hallmark of stem cells and cancer, allowed pinpointing two genetic programs, one of them expressed by embryonic stem cells (ESC), and the other by adult tissue stem cells. When analyzing expression of these programs in human cancers, it was observed that in tumors where the ESC-like transcriptional program was activated, strongly predicted metastasis and death, whereas expression of the adult tissue stem cells program led to a better prognosis (64). These types of analyses might allow to identify differentially expressed genes in the CSC as compared with the tumor mass and consequently be highly relevant for the identification of new CSC markers (cell surface markers, secreted proteins, intracellular proteins, or transcription factors). It should be noted that therapeutic antibodies can be generated, in addition to surface marker proteins, also against intracellular proteins, including transcription factors (65).

The next question that can be raised is whether the markers used for the identification of the CSC can also be used as therapeutic antibody targets. There is no straight answer to this question. Obviously, only mAbs that positively identify the CSC population could be used for therapeutic purposes. Returning to the example of the myelocytic leukemia, the combination of the CD34 and CD38 markers has been useful for the identification and isolation of the CSC (19). But since the CSCs are CD34^+^CD38^−^ cells, the CD38 antibody cannot be used for therapeutic purposes (the CSCs are negative for this marker), although the CD34 mAb could.

## Possible Strategies to Target CSC

**Figure 2:**
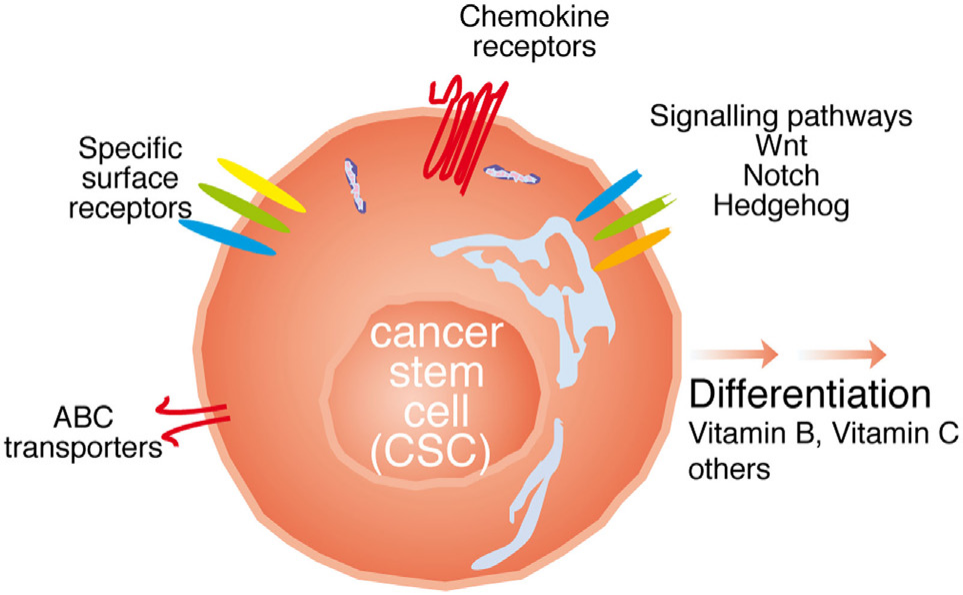
Possibilities to target cancer stem cell (CSC). The CSC can be targeted with either antibodies against specific surface receptors, interfering with signaling molecules relevant for CSC function such as Wnt, Notch, and Hedgehog, or the ATP-binding cassette (ABC) transporters, through chemokine receptor antibodies, or inducing the differentiation of thee cells.

*A priori*, the strategies to directly target the CSC population would tackle (i) differences in surface marker expression; (ii) interfere with signaling pathways relevant for their function; (iii) inhibit their function; (iv) interfere with metastasis formation; or (v) a combination of the above. In the following paragraphs, we will try to dissect these strategies (see Figure 2).

### Therapies Targeting CSC Surface Markers

It turns out, from the data on Table 1, that CD133 (prominin-1) has been established as a marker of CSC on many solid tumors including brain, colon, liver, lung, ovarian, pancreatic, and prostate tumors. The role of CD133 as a CSC marker has, however, been questioned, for example, using the lung cancer cell lines A549 and H446, where more than 45% of the cells represent bona-fide CSC, it has been reported that both CD133^+^ and CD133^−^ cells are able to form tumors with the same efficiency (29). In addition, CD133 exhibits several splice variants and different poorly characterized glycosylated isoforms (38), and as shown on Table 2, this antigen is broadly expressed on normal tissues (63). Thus, making it questionable whether CD133 represents a specific CSC marker and a therapeutic target for antibody-mediated elimination of CSC.

Targeting the adhesion molecule CD44 with monoclonal antibodies in xenografts of AML allowed to demonstrate that this treatment eradicated the leukemic CSC (66). Similarly, an antibody specific for the membrane IL-3Ra receptor (CD123) overexpressed in leukemia CSC (see Table 1) has been used to specifically target leukemia CSC in human AML. The treatments decreased leukemogenicity and eradicated CSC in mice (67, 68). In addition, an antibody targeting CD47 has demonstrated its ability to eliminate human acute lymphoblastic leukemia in xenograft transplants (69). The T cell immunoglobulin mucin-3 (TIM-3) was also identified as a surface molecule expressed on leukemia stem cells in most types of AML except for acute promyelocytic leukemia, but not on normal HSCs. TIM-3^+^ but not TIM-3^−^ AML cells engrafted, replicating in immunodeficient mice, many of the aspect on human AML. Furthermore, antibodies specific for TIM-3 dramatically diminished their leukemic burden (69). It should be noted that these experiments were carried out in xenotransplants, where the only cells expressing CD44, CD123, CD47, or TIM-3 were the transplanted tumor cells, thus any putative toxic effects on other body cells expressing these markers CD44^+^, CD123^+^, CD47^+^, or TIM-3^+^ could not be assessed in these models. However, a possibility, discussed in details in another review from this issue is to use combinations of antibodies (8), where even if the antibodies mentioned earlier for the treatment of AML used separately could be also toxic for the normal tissues, their combination (CD44, CD123, CD47, and TIM-3) could use smaller doses of each one of them, avoiding the concentrations required to induce toxicity in normal cells, but still be effective killing the CSC on AML. This is one of the possibilities that should be investigated for the treatment of AML and other types of cancer.

Another possibility of combination of antibodies against surface marker that can be investigated from the data on Table 1 deal with liver tumor CSCs, which are CD133^+^CD49f^+^CD90^+^ (48, 49). Each one of these markers is broadly expressed in normal tissues (63) as seen in Table 2. The use of antibodies against any of these markers as therapeutic tools might not be sufficiently selective for CSC and be toxic to healthy tissues. However, it might turn out that a strategy combining antibodies against the three molecules, using lower doses of each one of them, may still be effective while avoiding the unwanted toxicity with these lower doses.

Therapies targeting CSC surface markers can be exemplified by a clinical trial on untreated multiple myeloma using the anti-CD19 mAb MEDI-551 in combination with dexamethasone and lenalidomide. The rational of the trial is to determine whether the treatment with MEDI-551 decreases the number of CSC in these multiple myeloma patients (NCT01861340) (70).

In some cases, although the mAb identifies a target present on both adult stem cells and CSC, the antibody could be used to target the CSC. This would be the case for the mAb Nilo1, identifying mouse embryonic radial glia, adult neural stem cells, and also a subpopulation of mouse and human glioblastoma cells (71, 72), allowing to suggest that it might identify the CSC population (73). If Nilo1 indeed identifies the CSC, it could be envisaged that this antibody conjugated, for example, to gold nanoparticles would be able to photo-ablate Nilo1^+^ cells after these targeted cells absorb near infrared light. This would result in increased local temperature at the selected location, destroying the target cells (74). This approach would be feasible since the adult neural stem cells are restricted to their niche (subventricular zone), an expected different location from the tumor. However, in other tumor types, such as hematopoietic tumors or tumors of the mammary gland, this approach would be much more difficult to apply.

Another possible approach tackles the observation that both adult stem cell and CSC express higher levels of the ATP-binding cassette (ABC) transporters on their cell membranes. The ABC transporters have been proposed to contribute to multidrug resistance, because they allow to pump out of the cytoplasm many antitumor drugs, resulting in lower intracellular drug concentrations (35, 50), allowing the CSC to become more resistant to chemotherapeutic drugs (50, 75). However, some experiments using inhibitors of the ABC transporter have been successfully carried out (76). It seems that the generation of ABC transporter-blocking antibodies might inhibit ABC-transporter functions, without many of the negative toxic effects of the inhibitors, and therefore this will make the CSC more sensitive to chemotherapeutic drugs.

Finally, a clinical trial aims to determine the CSC load of HER2^+^ breast cancer tumors treated with the anti-HER2 antibody trastuzumab, in combination with adjuvant, doxorubicin hydrochloride, or cyclophosphamide followed by paclitaxel (NCT01424865).

### Targeting Signal Pathways

The signaling pathways involved in stemness, both in adult stem cells and CSC, including Notch, Hedgehog, and Wnt representing relevant therapeutic targets for CSC (9). Indeed, monoclonal antibodies against Notch are able to reduce the CSC population in colorectal tumors (77) and also in breast cancer cell lines (78). Similarly, antibodies against the Wnt-1 signaling pathway induce apoptosis in human colorectal cancer cells (79).

Small molecule Hedgehog antagonists have also been successfully used to inhibit systemic metastases in xenografts with tumors derived from human pancreas (80), but in this case, as far as the authors are aware, blocking antibodies have not yet been used. In fact, inhibitors of Wnt, Notch, and Hedgehog activities are being investigated in a clinical trial on esophageal cancer patients (NCT02221245). Other clinical trials use therapeutic antibodies against DLL4 to inhibit Notch signaling (presumably targeting Notch expressed on the CSC) in combination with paclitaxel in ovarian, peritoneal, and fallopian tube cancer (NCT03030287); in combination with FOLFIRI (irinotecan, folic acid, leucovorin, and fluorouracil), in metastatic colorectal cancer (NCT01189942); or the anti-DLL4 antibody demcizumab in combination with Gemcitabine Abraxane on metastatic pancreatic cancer (NCT01189929). Other examples use either a bispecific DLL4/VEGF antibody (OMP-305B83) in metastatic colorectal cancer, combined with the chemotherapeutic agents FOLFIRI (NCT03035253); or in combination with the chemotherapeutics carboplatin and pemetrexed for lung cancer (NCT01189968). Finally, another clinical trial uses the anti-DLL4 antibody demcizumab, in combination with the anti-PD-1 antibody pembrolizumab (immune checkpoint) in metastatic solid tumors (NCT02722954), aiming to inhibit Notch and simultaneously busting the antitumor immune response by inhibiting the PD-1/PD-L1 immune checkpoint.

A different approach used was to combine the Hedgehog inhibitor IPI-926 with the anti-EGFR antibody cetuximab in head and neck cancer patients (NCT01255800).

Other signaling pathways relevant in oncology include the tyrosine kinase family. The tyrosine kinase inhibitor lapatinib has been combined with the anti-HER2 antibody trastuzumab in a clinical trial in breast cancer patients (NCT00524303), where the authors want to analyze changes in CSC load.

### Trigger Differentiation

An additional possibility is to trigger the differentiation of the CSC. This will imply that they are not able to self-renew anymore, and therefore they would be more sensitive to regular chemotherapy and radiotherapy. In fact, several agents, such as retinoic acid (RA) [i.e., 13-*cis* RA (isotretinoin)], are used to modify cell expression patterns inhibiting proliferation and inducing cell differentiation and apoptosis (81–83). In addition, vitamin C has also been shown to trigger differentiation of CSC on leukemia, enhancing their sensitivity to PARP inhibition (84). It seems clear that these compounds will be used in combinations with antibodies and/or other drugs.

An example of therapeutic interest on triggering CSC differentiation is shown by a current clinical trial, aiming to analyze the role of the vitamin B derivate Fursultiamine on the differentiation of CSC in squamous cell carcinomas (NCT02423811) (76, 85).

### Others

The effects of any anti-CSC antibody can be potentiated if it is used in combination with antibodies inhibiting immune-checkpoints negative signals. These include antibodies binding to the PD-1 receptor on the T cells (nivolumab and pembrolizumab), to PD-L1 on the tumor cells (atezolizumab, durvalumab, and avelumab) or to CTLA-4 on T cells (ipilimumab) (86). This strategy will be relevant since the anti-checkpoint antibodies are able to switch the antitumor response from an immunosuppressed status, to another that allows to attack the tumor.

Since the CSCs are, in addition to the tumor-initiating cells, the unique cells that can form metastasis, as they are only cells within a tumor with a strong proliferation potential, able to generate the more differentiated tumor cells, which form the tumor mass, and at the same time a strong self-renewal potential through symmetric cell divisions (9). The use of any antibody or drug against the CSC, in combination with anti-chemokine receptor antibodies such as CXCR4, CCR7, and CCR9 (85, 87–96), would inhibit the migration of the CSC, their migration, invasion, and seeding of the metastatic cells, therefore improving the patient’s health.

Another possibility is to combine any antibody or drug specific for CSC with antibodies inhibiting tumor neo-vascularization, such as VEGF or VEGFR. In this context, there is a clinical trial that combines the preoperative treatment with the anti-VEGF antibody bevacizumab and chemotherapy in patients with breast cancer (NCT01190345), where they aim to determine the CSC activity (measured by the amount of aldehyde dehydrogenase 1/ALDH1^+^ cells before and after treatment).

Here, we have pinpointed some of the ongoing trials and preclinical experiments being carried out aiming to directly target CSC; however, there are many more possibilities to be carefully analyzed.

## Conclusion

The existence on many tumors of a subpopulation of cells with stem cell characteristics (the CSC population) it is clear by now. Furthermore, the concept that new anticancer treatments will be more effective if they directly target the CSC population, seems settled in the scientific community. The number of clinical trials targeting the CSC is, however, relatively small. Furthermore, from the 86 clinical trials found with the keywords “cancer stem cells,” only 12 of them use monoclonal antibodies as therapeutic agents. This is due, at least in part, to the lack of CSC-specific markers. We are optimistic, however, and believe that in the near future, this number will greatly increase. The new clinical trials will involve several combinations of antibodies, antibodies and chemotherapeutic drugs, small drug molecules, or the discovery of molecules able to differentiate the CSC. These will make a large advance in oncologic treatments specifically designed to destroy or kill CSCs.

Taken together, this does not mean that the work ahead will be easy, in particular since examples have been described where not only the CSC give rise to daughter CSC and non-CSC but also where the non-CSC population can, in some situations, give rise to some CSC (97). Thus, advances in the field of antibody immunotherapy directly targeting the CSC will require combinations of genetic analyses to identify differentially expressed genes in the CSC population, and an improved knowledge on the biology of the CSC (98), together with the use of complex algorithms to determine effective concentrations of different antibodies and drugs, to avoid adult stem cells harm. Thus, strategies using antibodies directly targeting the CSC population, while bursting the antitumor immune response and inhibiting neo-vascularization may represent an unparalleled opportunity to cure cancer.

## Author Contributions

All authors contributed to drafting, revising, and approving the final article.

## Conflict of Interest Statement

The authors declare that the research was conducted in the absence of any commercial or financial relationships that could be construed as a potential conflict of interest.
